# Prognostic Features of Recurrent Midline and H3 K27M-Mutant Glioma

**DOI:** 10.3390/cancers17132107

**Published:** 2025-06-23

**Authors:** Stephen J. Bagley, Yoshie Umemura, Joe S. Mendez, Isabel Arrillaga-Romany, Kevin J. Bielamowicz, Nick Butowski, Kelley Hutchins, Xiao-Tang Kong, Yazmin Odia, Akanksha Sharma, Lauren Weintraub, Carl Koschmann, Patrick Y. Wen, Amanda M. Saratsis, Tom Brundage, Samuel C. Ramage, Rohinton S. Tarapore, Truman Knowles, Dewen Yang, Joshua E. Allen, Timothy Cloughesy

**Affiliations:** 1 Perelman School of Medicine, University of Pennsylvania, Philadelphia, PA 19104, USA; sbagley@pennmedicine.upenn.edu; 2 Department of Neuro-Oncology, Barrow Neurological Institute, Phoenix, AZ 85013, USA; yoshie.umemura@barrowneuro.org; 3 Department of Neurosurgery, Huntsman Cancer Institute, University of Utah, Salt Lake City, UT 84112, USA; joe.mendez@hci.utah.edu; 4 Department of Neuro-Oncology, Massachusetts General Hospital, Boston, MA 02214, USA; iarrillaga@mgh.harvard.edu; 5 Department of Pediatrics, Arkansas Children’s Hospital, The University of Arkansas for Medical Sciences, Little Rock, AR 72202, USA; kjbielamowicz2@uams.edu; 6 Department of Neuro-Oncology, University of California, San Francisco, CA 94143, USA; nicholas.butowski@ucsf.edu (N.B.); truman.knowles@ucsf.edu (T.K.); 7 Department of Hematology/Oncology, Hawaii Pacific Health, Honolulu, HI 96813, USA; kelley.hutchins@hphmg.org; 8 Department of Neurology, University of California Irvine, Irvine, CA 92697, USA; xkong@hs.uci.edu; 9 Miami Cancer Institute, Miami, FL 33176, USA; yazmino@baptisthealth.net; 10 Department of Neuro-Oncology, Saint John’s Cancer Institute, Pacific Neuroscience Institute, Santa Monica, CA 90404, USA; ekakanksha@gmail.com; 11 Department of Pediatrics, Albany Medical Center, Albany, NY 12208, USA; weintrl@amc.edu; 12 Department of Pediatric Neuro-Oncology, University of Michigan, Ann Arbor, MI 48109, USA; ckoschma@med.umich.edu; 13 Center for Neuro-Oncology, Dana-Farber Center Institute, Boston, MA 02215, USA; patrick_wen@dfci.harvard.edu; 14 Department of Neurosurgery, Advocate Aurora Healthcare, Chicago, IL 60657, USA; amanda.saratsis@aah.org; 15 EB Enterprises, LLC, Chicago, IL 60634, USA; 16 Chimerix Inc., A Jazz Pharmaceuticals Company, Durham, NC 27713, USA; tbrundage@chimerix.com (T.B.); sramage@chimerix.com (S.C.R.); rtarapore@chimerix.com (R.S.T.); dqy44181@hotmail.com (D.Y.); jallen@chimerix.com (J.E.A.); 17 Department of Neurology, University of California, Los Angeles, CA 90095, USA

**Keywords:** H3 K27M, diffuse glioma, diffuse midline glioma

## Abstract

**Background/Objectives**: Midline glioma is a frequently morbid primary brain tumor often characterized by the histone mutation H3 K27M. The standard-of-care treatment is radiation therapy in the frontline setting, though effective treatment remains elusive and there is no established therapy in the second line or later setting. Here, we present the results of a multicenter, observational, retrospective study of the natural history of this disease in the recurrent setting when managed via standard-of-care interventions. **Methods**: Forty-four patients with recurrent H3 K27M-mutant and/or midline glioma after standard-of-care treatment were identified across 11 clinical centers in the United States who met inclusion criteria for evaluation. Data collected were analyzed by tumor radiographic appearance, age, anatomic location, and H3 K27M status, with factors contributing to overall survival (OS) identified. **Results**: Overall, median OS from time of first recurrence was 5.1 months (95% CI, 3.9 to 7.7%). In a subgroup analysis, survival was dismal across primary tumor locations, with a median OS of 3.7 months (95% CI: 0.7 to 9.8 months), 3.5 months (95% CI, 0.9 to not reached) for primary spinal, 5.1 months (95% CI to 0.2 not reached) for primary infratentorial, and 5.9 months (95% CI 4.4 to 14.7) for primary supratentorial tumors. In a multivariate analysis, DIPG and primary spinal tumor were associated with a higher risk of death. **Conclusions**: Taken together, these results shed light on prognostic factors and natural disease progression overtly related to recurrent midline and/or H3 K27M-mutant diffuse glioma, providing insight that can prove valuable for development of future clinical treatments for this recently defined disease.

## Simple Summary

Diffuse midline glioma is a malignant brain tumor with no effective treatment. These tumors often harbor a Histone H3 K27M mutation, associated with a more aggressive clinical course and poorer response to treatment. Standard-of-care treatment is radiation therapy, but disease typically recurs or progresses despite treatment and there is a paucity of the literature specific to the features and outcomes of recurrent disease. Given this, our group sought to explore what factors may be associated with disease progression and clinical outcomes to guide disease management and prognosis. Through a multicenter retrospective analysis of clinical data from patients with recurrent midline glioma and H3 K27M-mutant diffuse glioma, we identified features associated with poorer overall survival following progression following frontline therapy. Taken together, these data provide insight into tumor biology and clinical outcomes, potentially informing future clinical trials.

## 1. Introduction

High-grade glial tumors represent the most morbid form of brain cancer [1]. Approximately 80% of midline gliomas and 5% of cerebral hemispheric gliomas harbor a K27M mutation in the genes encoding isoforms of the Histone proteins H3.1 or H3.3 [2,3,4,5,6,7,8,9]. This somatic missense mutation is known as H3 K27M, and is associated with loss of H3 K27 trimethylation, poorer response to standard therapy, and lower overall survival, especially in younger patients [10]. To date, radiotherapy is the standard therapeutic intervention for diffuse midline gliomas, but only provides a median six-month event-free survival, with little effect on overall survival [5,10]. As such, the presence of an H3 K27M mutation in a midline tumor now confers a WHO Grade 4 status [11,12,13,14,15]. Indeed, if untreated, patients suffering from H3 K27M-mutant diffuse intrinsic pontine glioma (DIPG) have a median survival of only 1–4.5 months from diagnosis [6,16,17,18]. Further research has since revealed additional molecular characteristics of H3 K27M-mutant tumors, including distinct protein, RNA, DNA methylation, and epigenetic profiles [15]. This recent work has shed some light on a few novel prognostic factors in H3 K27M DMG, such as FGFR1 mutations that are associated with a more favorable clinical prognosis. However, there remains limited published information about prognostic factors and disease progression overtly related to the H3 K27M mutation in glioma [19], particularly in the recurrent setting. Therefore, we conducted a clinical history study that aimed to define outcomes and prognostic factors in patients with recurrent H3 K27M-mutant and/or midline glioma.

## 2. Materials and Methods

We performed a multicenter, retrospective, descriptive, observational study in patients with recurrent midline and/or H3 K27M-mutant glioma, to describe outcomes and prognostic factors associated with survival. Data were collected via retrospective chart reviews from eleven clinical centers across the United States (Supplemental Table S1); the protocol was approved by participating sites prior to study initiation. The clinical investigative site primary investigator was responsible for reviewing the medical records of potential patients. This retrospective, observational study was conducted with only the available medical records and imaging scans. All patient data collected were pseudonymized in the database. The primary objective for this analysis was to determine prognostic factors for overall survival (OS). Of note, this study was terminated by the sponsor prior to activation of all planned sites/countries and prior to achievement of complete enrollment in both cohorts. The early termination was based on revised regulatory agency feedback related to the utility of data from this study in regulatory decision-making, and resulted in lower patient enrollment than initially planned.

A potential patient was required to have met all the following criteria to be eligible for inclusion in the study:

1. Diagnosis of H3 K27M-mutant and/or midline glioma, initially diagnosed in 2012 or later.
2. Known tissue-proven H3 K27 status (H3 K27M-mutant or wild-type).
3. Medical records (including clinic notes and/or electronic databases) relating to glioma diagnosis and treatments received must be available for review. (Minimum information included demographics, disease characteristics, histology, disease history [diagnosis, tumor location, recurrences], radiation and other treatment history, survival status, and death date if applicable.)
4. Presence of recurrent disease after standard-of-care therapy.
5. No Prior Treatment with ONC201 or ONC 206.

### 2.1. Patients

**Figure 1:**
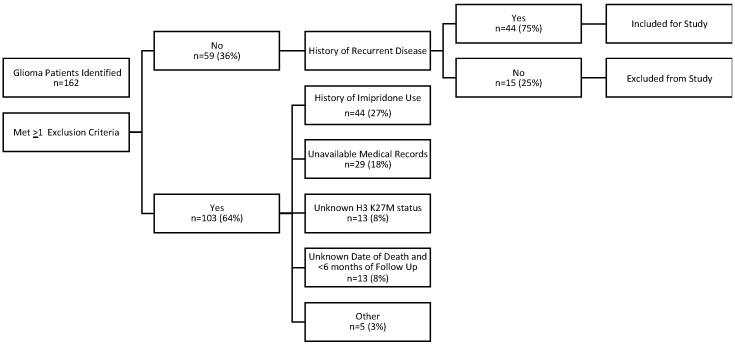
Patient selection flowchart citing inclusion and exclusion criteria and numbers ultimately yielding n = 44 patient records analyzed in the present study.

This study was intended to evaluate prognostic factors for survival in patients with biopsy-proven recurrent H3 K27M-mutant and/or midline glioma. A total of 162 potential patients were identified across participating institutions, with 44 patients with recurrent disease meeting the criteria for this analysis (Figure 1). Primary reasons for exclusion from analysis included treatment with ONC201 or ONC206 at any time (27.2%), insufficient medical records available (17.9%), unknown H3 K27M status (8%), and either an unknown date of death or <6 months survival follow-up from initial diagnosis (8%). One of the forty-four patients had no follow-up after first recurrence and therefore was censored as of first recurrence.

### 2.2. Evaluation and Statistical Methods

OS was estimated from first recurrence using the Kaplan–Meier (KM) method with median survival times and corresponding 95% confidence intervals (CIs) presented. KM survival estimates are presented at 12 and 24 months with corresponding 95% CIs. Multivariate Cox proportional hazard models were used to assess the impact of factors on survival. Final models following backward selection are presented with *p*-values, hazard ratios, and 95% CIs for each factor. It should be noted for some comparisons, analysis is exploratory in nature due to small sample size and as reflected by wide confidence intervals.

## 3. Results

Of the 162 glioma patient records reviewed, a total of 44 patients met the specified criteria and had evidence of recurrent/progressive disease (Figure 1): 30 patients (50.8%) with one recurrence, 9 patients (15.3%) with two recurrences, and 5 patients (6.8%) with ≥three recurrences. H3 K27M mutation was present in 68.2% of patients with recurrent disease (n = 30), with the remainder lacking the H3 K27M mutation (Table 1). Among the patients with the H3 K27M mutation, four (9.1%) patients had non-midline primary tumor locations, and the remainder had midline primary tumor locations. The median age of patients analyzed was 28 years (range 4 to 68 years) with 16 (36.4%) being less than 18 years of age. An even number of patients were male (n = 22) and female (n = 22), while the majority were White (68.2%) and not Hispanic or Latino (79.5%, Table 1).

All 44 patients analyzed had a tissue-proven diagnosis of glioma and received frontline radiation therapy as required for study eligibility. A total of 21 (47.7%) of these patients underwent a surgical procedure (sub, near, or gross total resection) in lieu of or in addition to a diagnostic biopsy. In addition to frontline radiation therapy, a wide range of anticancer agents were used as second-line therapy at the time of recurrence. The most common of these included temozolomide in 25 patients (56.8%), and bevacizumab in 7 patients (15.9%, Supplemental Table S2). Seven patients (11.9%) received re-irradiation during their disease course.

**Figure 2:**
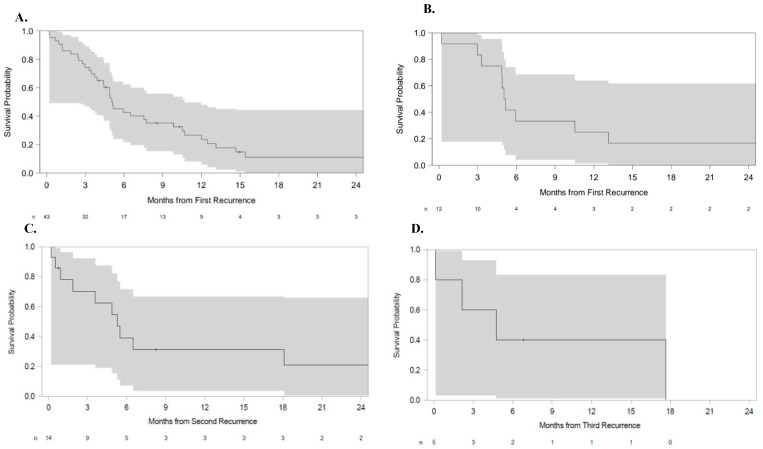
Overall survival (**A**) from first recurrence in all patients, (**B**) from first recurrence in patients excluding DIPG, leptomeningeal spread, CSF dissemination, or primary spinal tumors, (**C**) from second recurrence, and (**D**) from third recurrence. In general, OS was identical (5.1 months) from first recurrence in all patients, and in the cohort of patients excluding DIPG, leptomeningeal spread, CSF dissemination, and spinal tumors (**A** and **B**, respectively). Survival at second recurrence (5.3 months) was slightly longer than that at third recurrence (4.7 months, **C** and **D,** respectively). Shaded areas represent the 95% CI.

The median OS from the time of first recurrence was 5.1 months (95% CI: 3.9 to 7.7 months, Figure 2A). Excluding patients with DIPG, leptomeningeal spread, CSF dissemination, or primary spinal tumors (n = 12), the median OS was also 5.1 months (95% CI 3.0–13.1 months) (Figure 2B). Of the 14 patients reported to have two or more instances of recurrence, the median OS from second recurrence was 5.3 months (95% CI: 0.9 to 18.1 months) (Figure 2C). Of the remaining five patients with at least three instances of disease recurrence, the median OS from third recurrence was 4.7 months (95% CI: 0.1 to not reached) (Figure 2D).

Figure 2 shows patient survival after disease recurrence.

### 3.1. Analysis by H3 K27M Status

**Figure 3:**
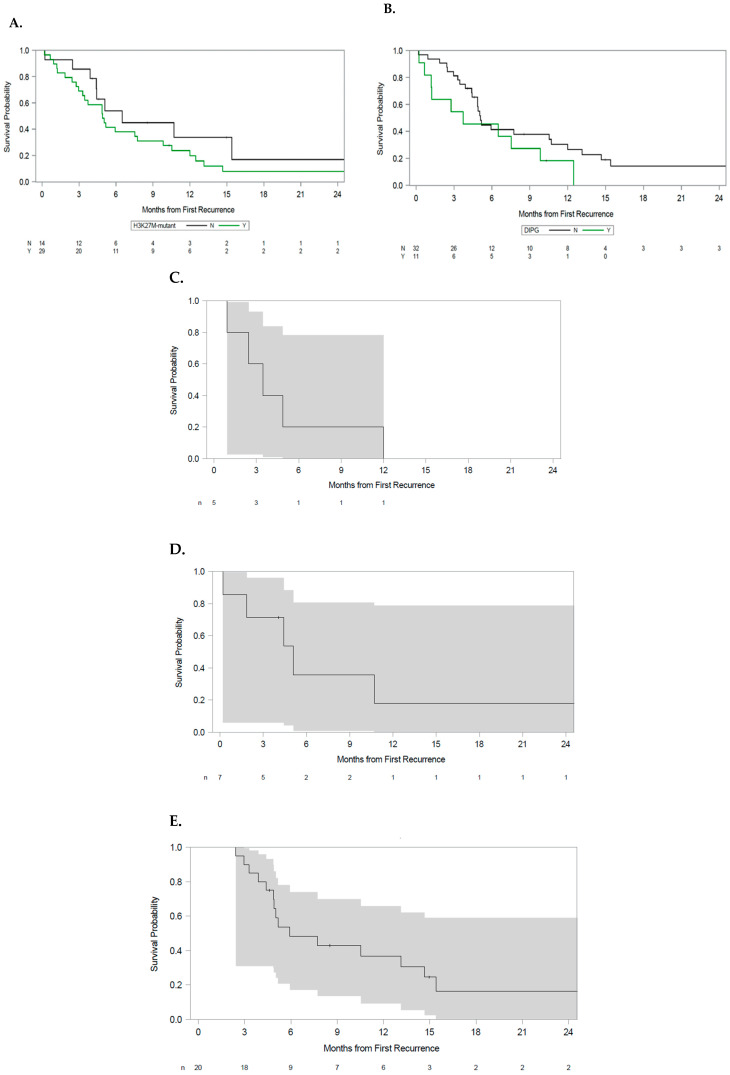
Overall survival by (**A**) H3 K27M mutation status, (**B**) DIPG versus other patients, (**C**) primary spinal tumors, (**D**) infratentorial tumors excluding DIPG, and (**E**) supratentorial tumors. Shaded areas represent the 95% CI.

Patients with the H3 K27M mutation had a median overall survival of 4.9 months (95% CI: 3.0 to 7.7 months). We observed a potential association between the presence of H3 K27M mutation (n = 30) and shorter overall survival; however, this relationship was not statistically significant (HR 1.8, 95% CI 0.8 to 3.8, *p* = 0.14, Figure 3A).

### 3.2. Analysis by Tumor Anatomic Origin

Patients with DIPG had a median OS from the time of first disease recurrence of 3.7 months (95% CI: 0.7 to 9.8 months). Patients with DIPG exhibited a trend toward worse survival compared to those patients without this diagnosis, although statistical significance was not reached (HR: 1.8, 95% CI 0.8 to 3.8, *p* = 0.14, Figure 3B).

Among patients with primary spinal tumors, overall survival for patients with primary spinal lesions from the time of first recurrence was 3.5 months (95% CI 0.9 to not reached, Figure 3C). Patients with infratentorial tumors had an OS of 5.1 months from the time of first recurrence (95% CI to 0.2 not reached Figure 3D). Patients with supratentorial lesions had an OS from the time of first disease recurrence of 5.9 months (95% CI 4.4 to 14.7, Figure 3E).

### 3.3. Multivariate Analysis

Multivariate Cox proportional hazard models conducted at first recurrence demonstrated that DIPG and primary spinal tumor were associated with a higher risk of death. HRs were 3.64 (95% CI: 1.41 to 9.45) and 4.69 (95% CI: 1.47 to 14.99), respectively (Table 2). Sub-total resections (n = 16) demonstrated a trend toward lowered risk of death (HR: 0.43; 95% CI: 0.19 to 1.01; *p* = 0.052). Due to the small sample size of patients with gross total resection (n = 2) and near gross resection (n = 3), the risk was inconclusive (Supplemental Figure S1).

## 4. Discussion

Diffuse midline glioma is a highly morbid primary brain tumor, and despite recent advances in our understanding of tumor biology and the study of more targeted therapies, effective treatment remains elusive. While standard-of-care interventions, including radiation +/− adjuvant therapy, are still the mainstay for DMG treatment, studies of novel approaches are currently underway for both initial and recurrent disease, critical efforts given the likelihood of disease progression and/or recurrence despite standard treatment.

For example, the role of re-irradiation and bevacizumab in the setting of recurrent high-grade glioma has been studied, with some reports suggesting improved OS [20]. Re-irradiation alone for recurrent DMG is also reported to show some survival benefit and improvement in neurologic deficits [21], but more studies are still needed to determine which patients will benefit from these additional therapies. Recent studies have also focused on more advanced and nuanced epigenetic features, including histone post-translational modification patterns and 3D chromatin structure, as well as molecular subtyping and prognostic modeling in H3 K27M gliomas [22,23,24]. These studies suggest novel potential biomarkers for prediction of treatment response and disease recurrence, and therefore warrant further investigation. Indeed, a recent study of adult glioma highlights the prognostic and predictive impact of key molecular alterations, such as IDH mutation and MGMT promotor methylation status [25], suggesting using a molecular approach to guide therapy is clinically relevant and feasible.

Yet despite these multiple ongoing and promising efforts, there remains a continued need to better understand the factors that drive treatment response and disease recurrence in patients with diffuse midline glioma. Here, we performed a multicenter, observational, retrospective exploratory study of the natural history of recurrent disease after management with standard-of-care interventions (radiation +/− adjuvant therapy). A variety of useful observations were made through the analysis of these data, many substantiating prior study findings of this tumor population, and others pointing to important factors for consideration of clinical management of these patients.

Firstly, a tumor with a pontine epicenter and diffuse appearance on MRI, consistent with diagnosis of DIPG, was associated with worse prognosis at first recurrence compared to tumors arising primarily in other anatomic locations. These data are in line with other reports suggesting that, when compared to patients with brainstem tumors, patients with other lesions such as in the thalamus experience significantly better survival [26]. As with previous reports in the literature, the presence of H3 K27M mutation in patients with recurrent disease was found to be associated with a trend toward worse OS in our study, though these findings were not statistically significant. The validity of this result is likely undermined by our study being underpowered, secondary to issues with achieving intended sample size and enrollment due to premature closure of the study. These factors likely impacted the statistical significance of the detected effect of H3K27M-mutant status on OS. To this end, Vuong et al. performed an extensive literature review comprising 26 studies and over 600 patients harboring H3.1 (n = 102) or H3.3 mutations (n = 529), finding that the prognosis of H3 K27M mutation in DMG patients is modulated by patient age, with poorer survival in children with the H3.3 mutation relative to adults [27]. Zheng et al. also performed a retrospective review of a series of pediatric and adult patients with diffuse midline gliomas harboring the H3 K27M mutation (n = 164), and found prognosis was better for adult patients than the pediatric cohort [28]. The results of these studies and our data suggest that the presence of H3 K27M mutation may be a greater prognostic factor when patient age is considered.

One particularly unique dataset arising from this study is the measure of OS in the setting of tumor recurrence. The median OS measured from first recurrence was 5.1 months. Importantly, prognosis at disease recurrence differed by disease location, with DIPG and primary spinal tumor associated with a higher risk of death after first recurrence. Due to the small sample size in this study, our analysis was not able to adequately determine any survival benefit or the risk of death after gross total resection (n = 2) versus near gross resection (n = 3). Other studies have shown that there is no survival benefit to greater extent of resection with midline gliomas [29,30]. In contrast, in the same study, preoperative KPS and adjuvant radiotherapy have been found to be independent clinical parameters influencing OS [29]. Taken together, these studies suggest that multiple factors must be considered when guiding patients regarding prognosis of recurrent disease.

Our study is not without limitations. Due to the early termination of the study, as described above in the Methods section, we did not achieve the full enrollment, with only 11 centers enrolling and all within the United States. The large difference in anticipated versus actual accrual limits the size of available subgroups in our subgroup analyses, and some multivariate analyses resulted in wide confidence intervals due to low numbers in comparison groups.

Another important point is that additional molecular features important to DMG biology have recently been discovered. For example, the current WHO classification now subsumes midline gliomas with H3 K27M mutations together with gliomas showing aberrant EZHIP expression or with an EGFR mutation. However, the analyzed specimens reported here were from patients initially diagnosed from 2012 to 2021, and hence not tested for molecular features like EZHIP or EFGR, which have since been discovered to be important in DMG biology.

## 5. Conclusions

There are limited data describing natural disease history and progression, as well as prognostic factors, in H3 K27M-mutant glioma, particularly in the recurrent setting. The outcomes described here, while exploratory in nature, shed some light on clinical outcomes and prognostic factors in the recurrent setting. The small sample size reported here highlights the crucial nature of multicenter collaborative studies for rare diseases. Much work is left to be performed to identify treatment modalities that alter the natural course of disease progression in H3 K27M-mutant glioma.

## Figures and Tables

**Table 1:** Patient demographic characteristics at first recurrence (N = 44) ^1^.

Demographic Feature	Number of Patients (%)
Age (years), median (range)	28 (4–68)
Pediatric, (age < 18 y)	16 (36.4%)
Female	22 (50%)
Race Asian Black White Multiple Not reported	5 (11.4%) 5 (11.4%) 30 (68.2%) 1 (2.3%) 3 (6.8%)
Ethnicity Hispanic or Latino Not Hispanic or Latino Unknown Not reported	7 (15.9%) 35 (79.5%) 1 (2.3%) 1 (2.3%)
Karnosfsky Performance Status (KPS) 100 90 80 70 60 50 40 Unknown	1 (2.3%) 3 (6.8%) 3 (6.8%) 10 (22.7%) 2 (4.5%) 3 (6.8%) 2 (4.5% 20 (45.5%)
H3 K27M mutation	30 (68.2%)
CSF dissemination *	5 (11.4%)
Leptomeningeal spread #	10 (22.7%)
Resection Gross total resection Near gross resection Sub-total resection None	2 (4.5%) 3 (6.8%) 16 (36.4%) 23 (52.3%)
Primary spinal tumor	5 (11.4%)
DIPG	12 (27.3%)
Multifocal disease †	12 (27.2%)
Number of recurrences 1 2 3 5	30 (68.2%) 9 (20.5%) 4 (9.1%) 1 (2.3%)
Contrast enhancement	36 (81.8%)
Steroid use (≥1.5 mg/day, dex)	18 (40.9%)

^1^ Key: Dex = dexamethasone; DIPG = diffuse intrinsic pontine glioma. * Defined as positive laboratory CSF cytology results. # Defined as radiographic evidence of additional disease in the CNS. † Defined as ≥2 lesions.

**Table 2:** Multivariate overall survival model, first recurrence final Cox model presented following backward selection, removing factors with *p* > 0.20. Improved overall survival HR < 1.0. Factors considered: sex, H3 K27M status, pediatric/adult, performance status, tumor size, resection, multifocal disease, DIPG/primary tumor location, contrast enhancement, steroid use, CSF dissemination, and leptomeningeal spread.

Parameter	Pr > ChiSq	Hazard Ratio	95% CI for Hazard Ratio
Primary tumor location: DIPG	0.0078	3.64	1.41–9.45
Primary tumor location: Non-DIPG brainstem	0.44	0.53	0.11–2.66
Primary tumor location: Non-DIPG spinal tumor	0.0092	4.69	1.47–14.99
Extent of resection: Sub-total resection	0.052	0.43	0.19–1.01
Extent of resection: Near gross resection	0.21	2.30	0.63–8.40
Extent of resection: Gross total resection	0.39	1.96	0.42–9.12

## Data Availability

Patient-level data collected as part of this analysis are not available for analysis by independent researchers. For more information, please contact clinicaltrials@chimerix.com.

## Supplementary Materials

The following supporting information can be downloaded at: https://www.mdpi.com/article/10.3390/cancers17132107/s1, Supplemental Table S1: Second-line therapies received. Table S2: Second Line Therapies Received. Supplemental Figure S1: Overall Survival after Disease recurrence. Hazard ratio with 95% Confidence Interval (CI) depicted.

## Appendix A

**PI Name**	**Institution**	**IRB**	**IRB Study Code**
Yazmin Odia	Miami Cancer Institute	Advarra IRB	SSU00180912
Kevin Bielamowicz	Arkansas Children’s Hospital	University of Arkansas for Medical Sciences IRB	274102
Lauren Weinbtraub	Albany Medical Center	Advarra IRB	SSU00180912
Xiao-Tang Kong	University of California, Irvine	University of California, Irvine Office of Research IRB	UCI IRB #769
Joe Mendez	The University of Utah, Huntsman Cancer Institute	The University of Utah IRB	IRB_00124158
Akanksha Sharma	Providence Saint John’s Health Center	Advarra IRB	SSU00180912
Yoshie Umemura	University of Michigan Health System	University of Michigan Medical School IRB	HUM00188956
Nicholas Butowski	University of California San Francisco	University of California San Francisco Human Research Protection Program IRB	257857
Isabel Arrillaga	Massachusetts General Hospital	Dana Farber Cancer Institute Office for Human Research Studies IRB	20–639
Stephen Bagley	University of Pennsylvania Pearlman Center for Advance Medicine	Office of the Institutional Review Board University of Pennsylvania	850834

## References

[1] Weller M., van den Bent M., Preusser M., Le Rhun E., Tonn J.C., Minniti G., Bendszus M., Balana C., Chinot O., Dirven L. (2020). EANO guidelines on the diagnosis and treatment of diffuse gliomas of adulthood. Nat. Rev. Clin. Oncol..

[2] Khuong-Quang D.-A., Buczkowicz P., Rakopoulos P., Liu X.-Y., Fontebasso A.M., Bouffet E., Bartels U., Albrecht S., Schwartzentruber J., Letourneau L. (2012). K27M mutation in histone H3.3 defines clinically and biologically distinct subgroups of pediatric diffuse intrinsic pontine gliomas. Acta Neuropathol..

[3] Schwartzentruber J., Korshunov A., Liu X.-Y., Jones D.T.W., Pfaff E., Jacob K., Sturm D., Fontebasso A.M., Khuong-Quang D.-A., Tönjes M. (2012). Driver mutations in histone H3.3 and chromatin remodelling genes in paediatric glioblastoma. Nature.

[4] Saratsis A.M., Kambhampati M., Snyder K., Yadavilli S., Devaney J.M., Harmon B., Hall J., Raabe E.H., An P., Weingart M. (2013). Comparative multidimensional molecular analyses of pediatric diffuse intrinsic pontine glioma reveals distinct molecular subtypes. Acta Neuropathol..

[5] Karremann M., Gielen G.H., Hoffmann M., Wiese M., Colditz N., Warmuth-Metz M., Bison B., Claviez A., van Vuurden D.G., O von Bueren A. (2017). Diffuse high-grade gliomas with H3 K27M mutations carry a dismal prognosis independent of tumor location. Neuro-Oncology.

[6] Erker C., Lane A., Chaney B., Leary S., E Minturn J., Bartels U., Packer R.J., Dorris K., Gottardo N.G., E Warren K. (2021). Characteristics of patients ≥10 years of age with diffuse intrinsic pontine glioma: A report from the International DIPG/DMG Registry. Neuro-Oncology.

[7] Aihara K., Mukasa A., Gotoh K., Saito K., Nagae G., Tsuji S., Tatsuno K., Yamamoto S., Takayanagi S., Narita Y. (2013). H3F3A K27M mutations in thalamic gliomas from young adult patients. Neuro-Oncology.

[8] Meyronet D., Esteban-Mader M., Bonnet C., Joly M.-O., Uro-Coste E., Amiel-Benouaich A., Forest F., Rousselot-Denis C., Burel-Vandenbos F., Bourg V. (2017). Characteristics of H3 K27M-mutant gliomas in adults. Neuro-Oncology.

[9] Ebrahimi A., Skardelly M., Schuhmann M.U., Ebinger M., Reuss D., Neumann M., Tabatabai G., Kohlhof-Meinecke P., Schittenhelm J. (2019). High frequency of H3 K27M mutations in adult midline gliomas. J. Cancer Res. Clin. Oncol..

[10] Lu V.M., Alvi M.A., McDonald K.L., Daniels D.J. (2019). Impact of the H3K27M mutation on survival in pediatric high-grade glioma: A systematic review and meta-analysis. J. Neurosurg. Pediatr..

[11] Pagès M., Beccaria K., Boddaert N., Saffroy R., Besnard A., Castel D., Fina F., Barets D., Barret E., Lacroix L. (2016). Co-occurrence of histone H3 K27M and BRAF V600E mutations in paediatric midline grade I ganglioglioma. Brain Pathol..

[12] Joyon N., Tauziède-Espariat A., Alentorn A., Giry M., Castel D., Capelle L., Zanello M., Varlet P., Bielle F. (2017). K27M mutation in *H3F3A* in ganglioglioma grade I with spontaneous malignant transformation extends the histopathological spectrum of the histone H3 oncogenic pathway. Neuropathol. Appl. Neurobiol..

[13] Mosaab A., El-Ayadi M., Khorshed E.N., Amer N., Refaat A., El-Beltagy M., Hassan Z., Soror S.H., Zaghloul M.S., El-Naggar S. (2020). Histone H3K27M Mutation Overrides Histological Grading in Pediatric Gliomas. Sci. Rep..

[14] Findlay I.J., De Iuliis G.N., Duchatel R.J., Jackson E.R., Vitanza N.A., Cain J.E., Waszak S.M., Dun M.D. (2021). Pharmaco-proteogenomic profiling of pediatric diffuse midline glioma to inform future treatment strategies. Oncogene.

[15] Solomon D.A., Wood M.D., Tihan T., Bollen A.W., Gupta N., Phillips J.J.J., Perry A. (2015). Diffuse Midline Gliomas with Histone H3-K27M Mutation: A Series of 47 Cases Assessing the Spectrum of Morphologic Variation and Associated Genetic Alterations. Brain Pathol..

[16] Langmoen I.A., Lundar T., Storm-Mathisen I., Lie S.O., Hovind K.H. (1991). Management of pediatric pontine gliomas. Child’s Nerv. Syst..

[17] Hoffman L.M., van Zanten S.E.V., Colditz N., Baugh J., Chaney B., Lane A., Fuller C., Miles L., Hawkins C., Bartels U. (2016). HG-75 Clinical, Radiological, and Histo-Genetic Characteristics of Long-Term Survivors of Diffuse Intrinsic Pontine Glioma: A Collaborative Report from the International and SIOP-E Dipg Registries. Neuro-Oncology.

[18] Baugh J., Colditz N., Janssens G., Dietzsch S., Hargrave D., von Bueren A., Kortmann R.-D., Bison B., van Vuurden D., van Zanten S.V. (2020). DIPG-77 Treatment Extent and the Effect on Survival in Diffuse Intrinsic Pontine Glioma. Neuro-Oncology.

[19] Schüller U., Iglauer P., Dorostkar M.M., Mawrin C., Herms J., Giese A., Glatzel M., Neumann J.E. (2021). Mutations within FGFR1 are associated with superior outcome in a series of 83 diffuse midline gliomas with H3F3A K27M mutations. Acta Neuropathol..

[20] Kulinich D.P., Sheppard J.P., Nguyen T., Kondajji A.M., Unterberger A., Duong C., Enomoto A., Patel K., Yang I. (2021). Radiotherapy versus combination radiotherapy-bevacizumab for the treatment of recurrent high-grade glioma: A systematic review. Acta Neurochir..

[21] Shariff N., Moreno A.S., Bennett J., Ramaswamy V., Das A., Liu A.P., Huang A., Tabori U., Hawkins C., Dirks P. (2025). Re-irradiation for children with diffuse intrinsic pontine glioma and diffuse midline glioma. Radiother. Oncol..

[22] Furth N., Algranati D., Dassa B., Beresh O., Fedyuk V., Morris N., Kasper L.H., Jones D., Monje M., Baker S.J. (2022). H3-K27M-mutant nucleosomes interact with MLL1 to shape the glioma epigenetic landscape. Cell Rep..

[23] Wang J., Huang T.Y.-T., Hou Y., Bartom E., Lu X., Shilatifard A., Yue F., Saratsis A. (2021). Epigenomic landscape and 3D genome structure in pediatric high-grade glioma. Sci. Adv..

[24] Bhattarai S., Hakkim F.L., Day C.A., Grigore F., Langfald A., Entin I., Hinchcliffe E.H., Robinson J.P. (2025). H3F3A K27M mutations drive a repressive transcriptome by modulating chromatin accessibility independent of H3K27me3 in Diffuse Midline Glioma. Epigenet. Chromatin.

[25] Saaid A., Monticelli M., Ricci A.A., Orlando G., Botta C., Zeppa P., Bianconi A., Osella-Abate S., Bruno F., Pellerino A. (2022). Prognostic Analysis of the IDH1 G105G (rs11554137) SNP in IDH-Wildtype Glioblastoma. Genes.

[26] Vuong H.G., Le H.T., Jea A., McNall-Knapp R., Dunn I.F. (2022). Risk stratification of H3 K27M–mutant diffuse midline gliomas based on anatomical locations: An integrated systematic review of individual participant data. J. Neurosurg. Pediatr..

[27] Vuong H.G., Ngo T.N.M., Le H.T., Dunn I.F. (2022). The prognostic significance of HIST1H3B/C and H3F3A K27M mutations in diffuse midline gliomas is influenced by patient age. J. Neuro-Oncol..

[28] Zheng L., Gong J., Yu T., Zou Y., Zhang M., Nie L., Chen X., Yue Q., Liu Y., Mao Q. (2022). Diffuse Midline Gliomas With Histone H3 K27M Mutation in Adults and Children. Am. J. Surg. Pathol..

[29] Wang Y., Feng L.-L., Ji P.-G., Liu J.-H., Guo S.-C., Zhai Y.-L., Sankey E.W., Wang Y., Xue Y.-R., Wang N. (2021). Clinical Features and Molecular Markers on Diffuse Midline Gliomas With H3K27M Mutations: A 43 Cases Retrospective Cohort Study. Front. Oncol..

[30] Manjunath N., Jha P., Singh J., Raheja A., Kaur K., Suri A., Garg A., Sharma M.C., Sarkar C., Mohan M. (2020). Clinico-pathological and molecular characterization of diffuse midline gliomas: Is there a prognostic significance?. Neurol. Sci..

