# Supplementary material for: Prognostic Features of Recurrent Midline and H3 K27M-Mutant Glioma
Source: Cancers (Basel). 2025 Jun 23;17(13):2107. doi: 10.3390/cancers17132107 (PMC12248830; doi:10.3390/cancers17132107)

Supplemental Material

Supplemental Table S1: Participating Medical Centers.

Medical Center	Location
University of Pennsylvania	Philadelphia, PA, USA
University of Michigan	Ann Arbor, MI, USA
University of Utah/Huntsman Cancer Institute	Salt Lake City, Utah, USA
Massachusetts General Hospital	Boston, MA, USA
The University of Arkansas for Medical Sciences/Arkansas Children's Hospital	Little Rock, AR, USA
University of California	San Francisco, CA, USA
Hawaii Pacific Health	Honolulu, HI, USA
University of California Irvine	Irvine, CA, USA
Miami Cancer Institute, Baptist Health South Florida	Miami, FL, USA
Pacific Neuroscience Institute/Saint John's Cancer Institute	Santa Monica, CA, USA
Albany Medical Center	Albany, NY, USA
Dana-Farber/Brigham and Women's Cancer Center	Boston, MA, USA

Supplemental Table S2: Second Line Therapies Received. Number of patients receiving indicated anticancer therapy (patients with at least one intervention in addition to frontline radiation therapy).

ABI-009 (NAB-RAPAMYCIN)	1
ADJUVANT TRANS SODIUM CROCETINATE (TSC) - CLINICAL TRIAL	1
BELINOSTAT	1
BEVACIZUMAB	7
CARBOPLATIN	1
CONCURRENT TRANS SODIUM CROCETINATE (TSC) - CLINICAL TRIAL	1
ETOPOSIDE	1
EVEROLIMUS	3
IPILIMUMAB	1
IRINOTECAN	1
LOMUSTINE	1
NAB-RAPAMYCIN	1
NILOTINIB	1
NIVOLUMAB	1
OPTUNE	3

PALBOCICLIB	1
PANOBINOSTAT	2
PAZOPANIB	1
PONATINIB	1
TEMOZOLOMIDE	25
TEMSIROLIMUS	1
TRAMETINIB	3
VORINOSTAT	1

Supplemental Figure S1. Overall Survival after Disease recurrence. Hazard ratio with 95% Confidence Interval (CI) depicted

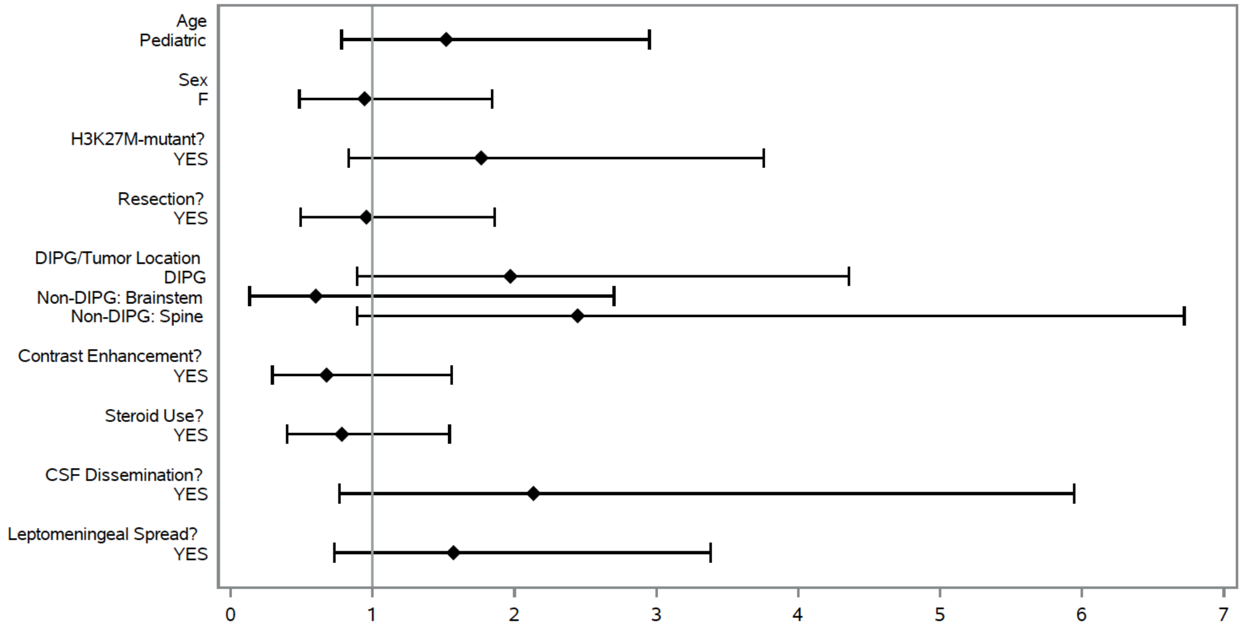

Supplement: Supplementary file 1 [file cancers-17-02107-s001.zip › cancers-3624729-supplementary.pdf]
